# Fabrication and Characterization of Iridium Oxide pH Microelectrodes Based on Sputter Deposition Method

**DOI:** 10.3390/s21154996

**Published:** 2021-07-23

**Authors:** Ye Xi, Zhejun Guo, Longchun Wang, Qingda Xu, Tao Ruan, Jingquan Liu

**Affiliations:** National Key Laboratory of Science and Technology on Micro/Nano Fabrication Laboratory, Collaborative Innovation Center of IFSA, Department of Micro/Nano-Electronics, Shanghai Jiao Tong University, Shanghai 200240, China; yesiki@sjtu.edu.cn (Y.X.); gzj98762@sjtu.edu.cn (Z.G.); LongchunWang@sjtu.edu.cn (L.W.); qdxu0830@sjtu.edu.cn (Q.X.); ruantao@sjtu.edu.cn (T.R.)

**Keywords:** pH electrode, iridium oxide, long-term stability, near-Nernstian response

## Abstract

pH value plays an important role in many fields such as chemistry and biology; therefore, rapid and accurate pH measurement is very important. Because of its advantages in preparation, wide test range, rapid response, and good biocompatibility, iridium oxide material has received more and more attention. In this paper, we present a method for preparing iridium oxide pH microelectrodes based on the sputter deposition method. The sputtering parameters of iridium oxide are also studied and optimized. Open-circuit potential tests show that microelectrodes exhibit near-Nernstian pH response with good linearity (about 60 mV/pH), fast response, high stability (a slight periodic fluctuation of potential change <2.5 mV in 24 h), and good reversibility in the pH range of 1.00–13.00.

## 1. Introduction

pH is a fundamental parameter in many fields ranging from agriculture, husbandry, and chemical engineering to biology and medicine; therefore, rapid and accurate pH measurements are especially important [1,2,3,4,5,6,7,8]. For example, the growth of plants is closely related to pH, so it is important to measure the pH of soil and agricultural water for growth management. Chemical processes are also closely related to pH. Choosing the correct pH for an aqueous solution can effectively avoid unwanted reactions. For living organisms, metabolic processes can occur only when specific physical and chemical conditions are met, which is why the pH of extracellular fluids is maintained at 7.4 by an effective buffer system [5]. At present, the most widely used pH sensor is the glass pH electrode. However, due to the characteristics of the glass itself, the glass pH electrode has many shortcomings, including high input impedance, vulnerability, incompatibility with HF solutions, and large errors in alkaline solutions. In addition, glass electrodes cannot be miniaturized, which limits their applications in microscale environments and in vivo online monitoring [9,10]. In recent years, researchers have been working on the development of novel pH microsensors, in the context of which metal/metal oxide electrodes have received more and more attention. Among these metal oxide electrode materials, iridium and its oxides have attracted the most attention because they have advantages in processing, micromanipulation, wide test range, rapid response time, and good biocompatibility [11,12,13,14,15,16,17,18]. These characteristics make it suitable for extreme environments and in vivo pH measurement. The iridium oxide layer can be prepared by the electrodeposition method, electrochemical cyclic voltammetry method, thermal oxidation method, sputtering deposition method, etc.

So far, the methods most used to prepare iridium oxide thin films are the electrodeposition method and electrochemical cyclic voltammetry method. The electrochemical deposition method deposits iridium oxide thin films on graphite, titanium, platinum, and other conductor substrates in the electroplating solution under a certain current. This method does not need iridium metal as the substrate material and is suitable for the preparation of microelectrodes, but the process conditions are difficult to control. The preparation of iridium oxide films by electrochemical cyclic voltammetry is carried out in an electrolyte solution of sulfuric acid or sodium hydroxide. During the preparation process, the metal iridium is electrochemically activated, and a thin layer of iridium oxide grows on the surface of pure iridium. This method requires pure iridium as the substrate material, and the preparation process needs to be carried out in a strong acid or base solution. These limit the application of this method to the preparation of microelectrodes or ultramicroelectrodes. At present, the thermal oxidation methods mainly include the thermal decomposition of IrCl_3_, the lithium carbonate oxidation sintering method, and the direct burning method. Iridium oxide thin films prepared by thermal oxidation have good adhesion and stability; this is thus an ideal method for the preparation of iridium oxide thin films. However, this method also needs specific materials as the substrate material and needs to be prepared in a high temperature environment of 400–900 degrees Celsius. It is also difficult to miniaturize the samples.

Compared to the previous methods, the E-pH response sensitivity of iridium oxide thin films prepared by sputtering deposition method is close to the Nernst response, and the properties of the prepared thin films have good reproducibility. This method requires no specific material as a substrate and can be used to prepare micro-scale sensors in combination with photolithography and other technologies. These characteristics enable this method to be applied to microelectrodes and multifunctional lab-on-chip type systems. In this work, we focused on the preparation of iridium oxide microelectrodes by the sputter deposition method because the sputter deposition method is suitable for the miniaturization of sensors and has fewer preparation constraints. We also presented a method for preparing the iridium oxide pH microelectrode. The sputtering parameters of iridium oxide were studied and optimized. Finally, we tested the key performance characteristics (response time, long-term stability, reversibility, and sensitivity) of the iridium oxide pH microelectrodes prepared with optimized parameters.

## 2. Materials and Methods

### 2.1. Materials

Sodium hydroxide (NaOH), hydrochloric acid (HCl), potassium chloride (KCl), acetone (CH_3_COCH_3_), ethanol (C_2_H_5_OH), and pH damping fluids with different pH values were purchased from Sinopharm Chemical Reagent Company, Ltd. (Shanghai, China). All chemicals used in the experiments were of reagent grade quality or better and were used without further purification. Aqueous solutions were prepared from de-ionized water.

Borosilicate glass tubes (o.d. = 1.0 mm, i.d. = 0.58 mm) were purchased from Sutter Instrument Company. Fine grade 800-grit sandpapers were purchased from Zhejianglixie Instrument Company, Ltd. (Yiwu, China). Apiezon wax was purchased from M&I Materials Ltd. (Manchester, UK).

### 2.2. Instruments

A laser-based puller system (P-2000, Sutter Instrument Company, Novato, CA, USA) was used in the fabrication of the micropipettes. An electrode beveler (BV-10, Sutter Instrument Company, Novato, CA, USA) with a home-made clamp was used in the polishing process. Electrochemical testing was carried out on a CHI 660c electrochemical workstation (CH Instrument, Austin, TX, USA). A high vacuum scanning electron microscope (ULTRA55, Zeiss, Oberkochen, Germany) was used for SEM observation and energy dispersive spectrometry (EDS).

### 2.3. Preparation of Iridium Oxide pH Microelectrode

**Figure 1:**
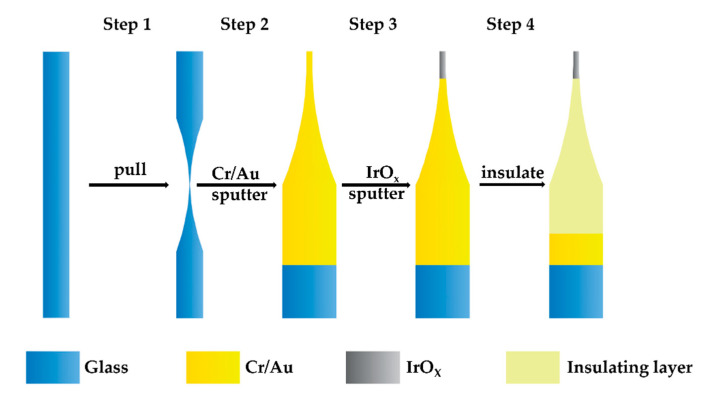
Process diagram showing the preparation of iridium oxide pH microelectrode based on sputter deposition method.

Figure 1 shows a four-step process for fabrication of iridium oxide pH microelectrodes. At first, borosilicate glass tube was ultrasonically cleaned in acetone, ethanol, and de-ionized water and dried in a drying oven. In this step, a laser-assisted pulling method was adopted to prepare the micropipettes. The P-2000 laser-based micropipette puller system was used in the pulling process. The adjustment parameters for the puller system were as follows: heat, velocity, delay, pull, and filament [19]. The shape of the micropipettes was controlled by these five parameters. Optimized by experiments, the borosilicate glass tube was pulled into two micropipettes under the following parameters: heat = 420, filament = 4, velocity = 40, delay = 130, pull = 140. Then the tip of micropipette was polished to a smooth cross section with the electrode beveler and 800-grit sandpaper. The polished micropipettes were subsequently ultrasonically cleaned in acetone, ethanol, and de-ionized water and dried in a drying oven.

In the second step, the micropipettes were splashed with a Cr/Au conductive layer in a custom-made reactive sputtering apparatus. The thickness of this mixed metal layer was about 200 nm, and the Cr layer was located between the glass and the Au layer to improve the bonding rate. It is worth noting that because the micropipettes were sputtered on a flat tray, sputtering was required twice (on the front and back) to ensure a complete coating of the metal layer.

In the third step, the front ends of the micropipettes were splashed with an iridium oxide layer in a custom-made reactive sputtering apparatus. During the sputtering process, a hard mask was used to achieve local iridium oxide sputtering on the micropipette tips. As in the second step, sputtering was required twice, to cover both the front and back sides.

In the fourth step, the iridium oxide pH microelectrodes were prepared by insulating and wiring the micropipettes. The Apiezon wax was first melted by heating on a hot plate at 200 °C [20]. Under the control of a micromanipulator, each micropipette was then passed through the liquid Apiezon wax. The micropipettes coated with Apiezon wax at the front were then heat-treated in an oven at 90 °C for 5 min and the very tips of the iridium oxide layer were exposed after the process [21]. Finally, iridium oxide pH microelectrodes were prepared by connecting a silver wire with conductive silver paste on the gold layer at the tail end.

### 2.4. Electrochemical Testing

**Figure 2:**
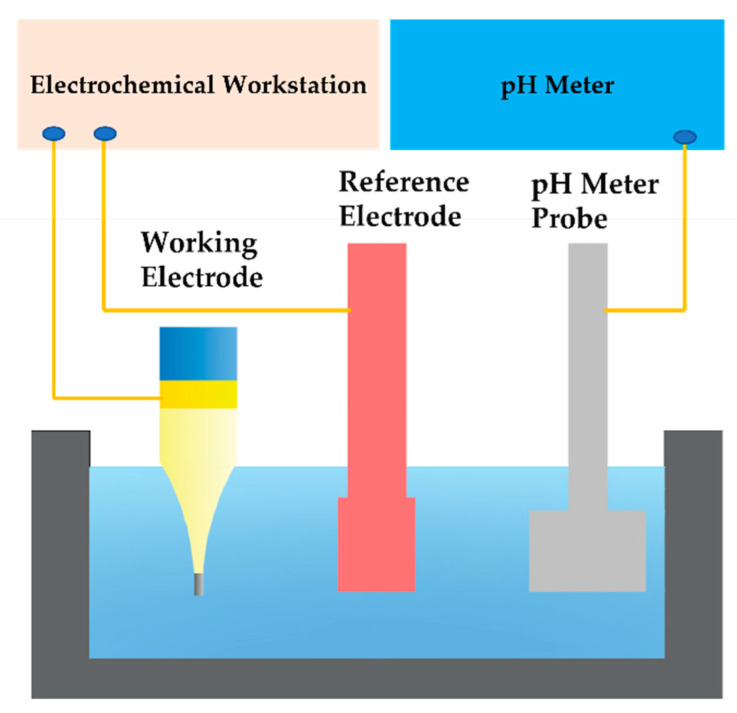
Schematic diagram of electrochemical test.

Open-circuit potentials of iridium oxide pH microelectrodes were tested against a silver chloride reference electrode in pH buffer solution in a clean room, which was maintained at about 20 °C. The potentials of the iridium oxide pH microelectrodes were measured with an electrochemical workstation (CHI 660c, CH Instrument, Austin, TX, USA). As shown in Figure 2, the electrochemical tests all adopted a double electrode structure, and a pH meter was always used for calibration during the tests. The response time, long-term stability, reversibility, and sensitivity of the iridium oxide pH microelectrodes were examined. In pH sensitivity experiments, measurements were carried out in a series of commercial pH buffer solutions with pH values of 4.00, 6.86, and 9.18, respectively; the solutions were air-saturated without stirring. In the pH response time and long-term stability experiments, measurements were carried out in commercial pH buffer solutions with a pH value of 7.00. In reversibility experiments, measurements were carried out in a series of commercial pH buffer solutions with pH values of 1.00, 4.00, 7.00, 10.00, and 13.00, respectively, and the pH change was realized by switching the iridium oxide pH microelectrodes from one pH buffer solution to another.

## 3. Results and Discussion

### 3.1. Preparation of Iridium Oxide Layer

The preparation of the iridium oxide layer was based on the sputtering deposition method because the response sensitivity of the iridium oxide layer prepared by this method was close to the Nernst response of 59 mV/pH, and the response results had high reproducibility. In this method, argon (Ar) was generally used as the feed gas and oxygen (O_2_) was added to the feed (carrier) gas. The partial pressure of O_2_ determined the mixing ratio of metal and oxide in the sputtered sediments. In addition, the partial pressure of working gas, temperature, humidity, and power supply also had important effects on the response sensitivity, stability, and response time of the iridium oxide layer. In this study, we used a custom reactive sputtering machine to prepare the iridium oxide layer, and the entire preparation process was performed in a purification room with stable temperature and humidity. Considering that the temperature and humidity remained stable, the influence of the partial pressure of the working gas and sputtering time on the sputtering iridium oxide layer were mainly studied under the condition of sputtering power of 100 W.

**Figure 3:**
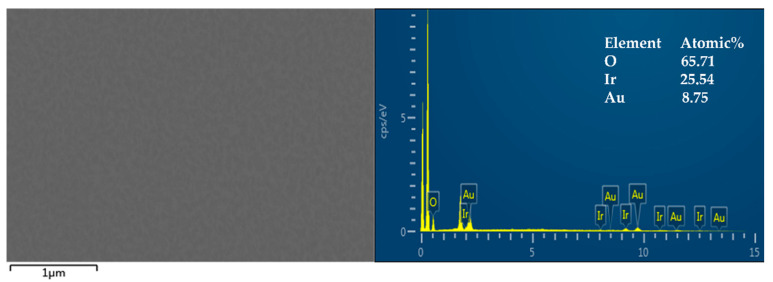
The surface morphology and EDS of iridium oxide film; insets are the atomic percentage of different elements.

The sputtering of iridium oxide films was mainly divided into two steps: iridium seed layer sputtering and iridium oxide layer sputtering. The experimental results proved that seed layer sputtering had two advantages that could effectively improve the stability of subsequent sputtering of iridium oxide film and increase the bonding rate between the iridium oxide layer and gold layer. The sputtering of the iridium seed layer was carried out in pure argon at 10 sccm, and its sputtering power was 100 W. The typical sputtering time for the iridium oxide seed layer was 2 min. As shown in Figure 3, the prepared iridium oxide film was tested by EDS, and the results showed the percentages of oxygen, iridium, and gold atoms in the film to be 65.71%, 25.54%, and 8.75%, respectively. Considering that the iridium oxide film was prepared on a gold layer, the results showed that the film of iridium oxide had been successfully deposited using the sputtering deposition method.

**Figure 4:**
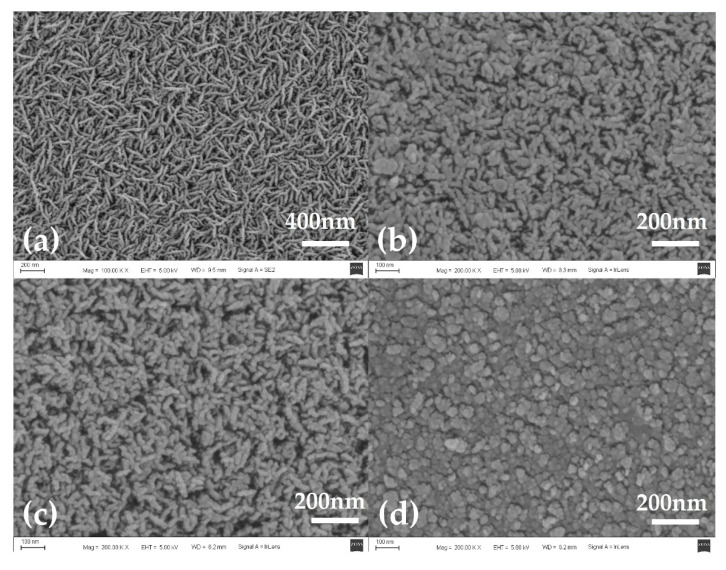
The surface morphology of iridium oxide films prepared at oxygen flow rates of (**a**) 10 sccm, (**b**) 20 sccm, (**c**) 30 sccm, and (**d**) 40 sccm; the Ar flow rate was maintained at 10 sccm during preparation, and the sputtering time was 15 min.

After repeated experiments, the results showed that the iridium oxide layer could be stably prepared by adding a certain flow of O_2_ when the flow of Ar was set to 10 sccm. Figure 4 shows the surface morphology of iridium oxide films prepared at O_2_ flow rates of 10 sccm, 20 sccm, 30 sccm, and 40 sccm, respectively. The Ar flow rate was maintained at 10 sccm during film preparation, and the sputtering time was 15 min.

The sputtering deposition method could be used to fabricate various structured iridium oxide films under different preparation parameters such as working atmosphere, temperature, humidity, etc. [22]. The following structures of iridium oxide film have been obtained: nanowire arrays, nanoplatelet arrays, nano foils, nanolayers of core-shell and 1D nanocrystals, etc. [11]. In this study, the prepared iridium oxide films exhibited a typical nanowire array structure as shown in Figure 4a–c. When the working gases were 10 sccm of O_2_ and Ar respectively, the resulting iridium oxide nanowires were very thin, with a length of about 200 nm and a diameter of less than 30 nm. These nanowires were uniformly distributed throughout the sample surface. With the increase of oxygen flow, the length of the nanowires remained unchanged, but the diameter of the nanowires increased significantly. When the oxygen flow was increased to 40 sccm, the iridium oxide nanowires disappeared, and the iridium oxide film appeared as a planar structure composed of many particles as shown in Figure 4d. These particles could be thought of as the products of nanowires when their diameters were sufficiently increased.

**Figure 5:**
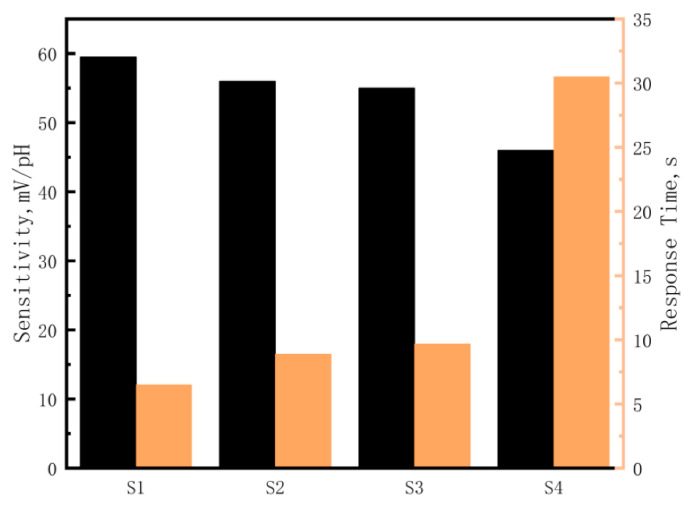
The sensitivity and response time of iridium oxide films prepared at oxygen flow rates of (**a**) S1 10 sccm, (**b**) S2 20 sccm, (**c**) S3 30 sccm, and (**d**) S4 40 sccm; the Ar flow rate was maintained at 10 sccm during the sputtering process.

The sensitivity and response times of iridium oxide films prepared at different oxygen flow rates were as shown in Figure 5. Through the linear fitting calculation of open-circuit potential values in commercial pH buffer solutions with pH values of 4.00, 6.86, and 9.18, the sensitivity of S1–4 was 59.5 mV/pH, 56.1 mV/pH, 55.0 mV/pH and 46.2 mV/pH, respectively. The response time of the microelectrode was measured in commercial pH buffer solution with a pH value of 7.00, and the response time of the four samples was 6.5 s, 8.9 s, 9.7 s, and 30.5 s, respectively. The results showed that the sensitivity of the iridium oxide microelectrodes decreased, and the response time increased, with increases in oxygen flow. Combined with the surface morphology of iridium oxide film in Figure 4, this phenomenon was caused by the increase in the diameter of iridium oxide nanowires because the specific surface area of the film decreased continuously and the iridium oxide in the inner layer of the film could not react with the solution effectively and quickly as the diameter of iridium oxide nanowires gradually increased. So, the oxygen flow rate of 10 sccm and argon flow rate of 10 sccm were selected as parameters for the preparation of iridium oxide microelectrodes in this study.

**Figure 6:**
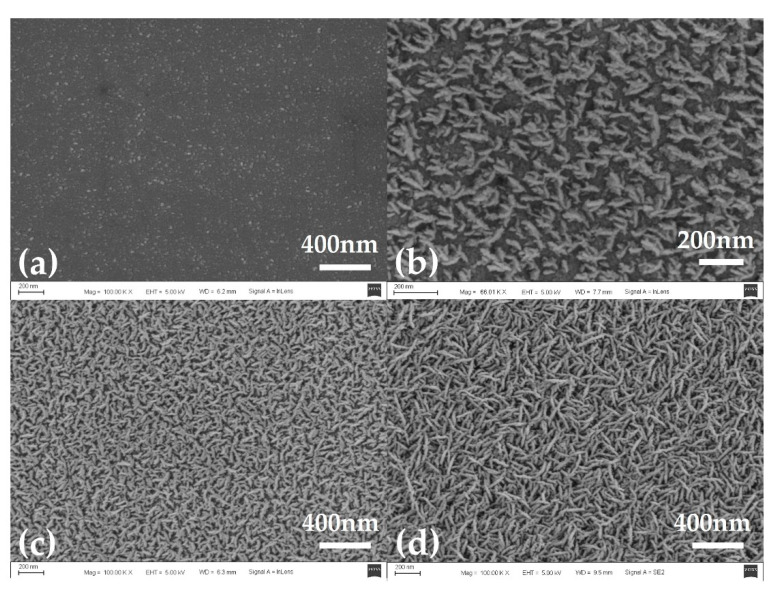
The surface morphology of iridium oxide films prepared with a sputtering time of (**a**) 1 min, (**b**) 3 min, (**c**) 5 min and (**d**) 15 min; the oxygen and argon flow rates were all maintained at 10 sccm during the sputtering process.

The effect of different sputtering times on the morphology of the sample was also studied. As shown in Figure 6a, there were a large number of tiny particles on the surface of the substrate. When the sputtering time reached 3 min, the iridium oxide nanowires began to appear. At this point, the iridium oxide nanowires were relatively sparse, and the substrate was still clearly visible. As the sputtering time increased, iridium oxide nanowires eventually covered the entire substrate, but they were short in length. After sputtering for 15 min, the iridium oxide nanowires were uniformly distributed throughout the substrate and significantly increased in length, which is shown in Figure 6d. The iridium oxide film in Figure 6d shows a more three-dimensional structure than that in Figure 6c, and that was beneficial for pH sensing. Hence, the sputtering time of 10 min was selected as the preparation parameter for fabrication of iridium oxide microelectrodes in this study.

### 3.2. PH Sensing of Iridium Oxide Microelectrodes

**Figure 7:**
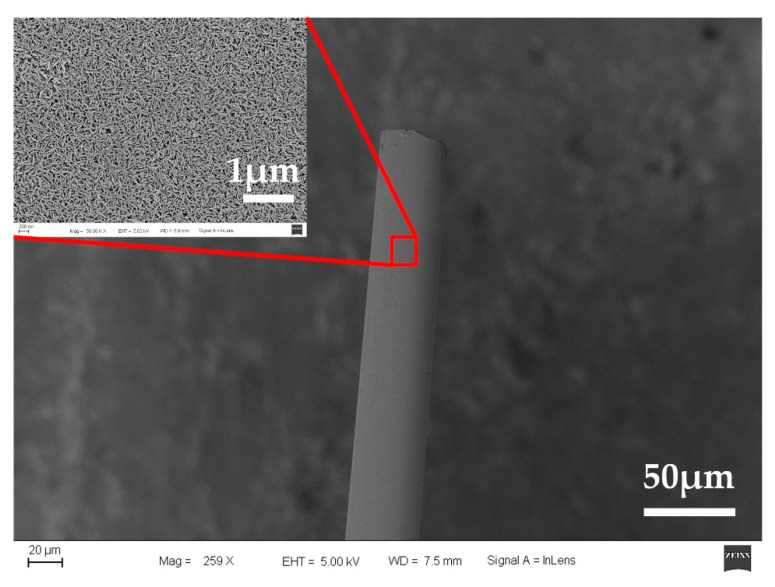
The morphology of an iridium oxide microelectrode under the following parameters: 10 sccm O_2_, 10 sccm Ar, and 15 min of sputtering time.

As shown in Figure 1, we prepared the iridium oxide microelectrodes under the following parameters: 10 sccm O_2_, 10 sccm Ar, and 15 min of sputtering time. One resulting iridium oxide pH microelectrode is shown in Figure 7. The diameter of the microelectrode was about 40μm, and the tip of the microelectrode was sputtered with a dense iridium oxide film.

**Figure 8:**
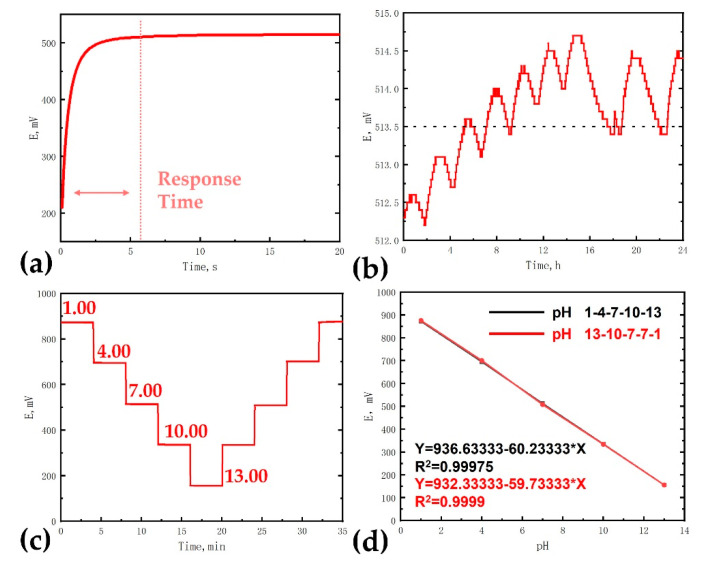
The measurement of pH sensing of iridium oxide pH microelectrodes: (**a**) response time, (**b**) potential stability test, (**c**) potential response to pH changes for acid-to-base and base-to-acid, (**d**) potential-pH dependence for acid-to-base and base-to-acid.

The response time, long-term stability, sensitivity, and reversibility of the iridium oxide pH microelectrodes were examined as shown in Figure 8. Different from the voltammetry pH test, the open circuit potential test of microelectrodes in solution could quickly and conveniently obtain pH test results.

The response time was first measured in commercial pH buffer solutions with a pH value of 7.00, as shown in Figure 8a. According to the International Union of Pure and Applied Chemistry (IUPAC), the electrode can be considered to reach a stable state when the rate of change of electrode potential is less than 1 mV/min. In this study, the time required for the electrode to reach a steady state was defined as the response time. The average response time was 5.9 s in multiple sample tests (N = 5). The rapid response could meet the criteria for biological pH sensing, such as blood pH sensing, cell culture pH sensing, etc.

Long-term stability was another important index to evaluate the performance of pH electrodes. A large drift of potential would severely limit the application of pH electrodes, especially in monitoring in-situ signals where it is difficult to perform quadratic calibration. The long-term stability experiment was carried out in commercial pH buffer solutions with a pH value of 7.00. The test environment was a clean room with constant temperature and humidity, and the test time was 24 h. As shown in Figure 8b, the potential of the iridium oxide pH microelectrode was between 512.2 mV and 514.7 mV in 24 h. The potential tended to fluctuate within a small range. The long-term stability of iridium oxide films prepared by sputtering deposition was greatly affected by the preparation process. In general, iridium oxide films prepared by electrochemical deposition and electrochemical cyclic voltammetry have better long-term stability because the films prepared by these two methods are hydrated. The long-term stability of iridium oxide films in this paper was close to the performance of iridium oxide films prepared by electrochemical deposition [13]. This measurement error was well within the acceptable range for most application cases.

To fully evaluate the pH-sensing and reversibility characteristics of the iridium oxide pH microelectrode, the potential response to pH changes in three steps between pH 1.00–13.00 was measured; the results are shown in Figure 8c. The potential was continuously measured for 5 min for each pH value. Almost identical variations in potential were observed for pH changes from lower (e.g., pH 1 to 4) or higher (e.g., pH 7 to 4) pH values.

Two good linear correlations were observed in Figure 8d. The potential response to pH changes for acid-to-base and base-to-acid both presented good linear relationships and reversibility. The sensitivities to pH 1–4–7–10–13 and pH 13–10–7–4–1 were 60.23 mV/pH and 59.73 mV/pH, which were close to the theoretical Nernst value (57.80 mV/pH). The results exhibit the characteristic sensitivity of typical non-hydrated films and are similar to those of iridium oxide films prepared by thermal oxidation and sputtering deposition [15]. The results demonstrate that the iridium oxide microelectrode maintained stable pH sensitivity and reversibility over a wide pH range (pH 1–13).

## 4. Conclusions

In this study, we investigated the preparation parameters of the sputtering deposition method, such as oxygen flow rate and sputtering time, and also optimized the preparation parameters for iridium oxide film. The iridium oxide microelectrodes based on the sputtering deposition method were successfully fabricated under the following parameters: 10 sccm O_2_, 10 sccm Ar, and 15 min of sputtering time. The response time, long-term stability, sensitivity, and reversibility of the iridium oxide pH microelectrodes were examined. The results indicated that iridium oxide pH microelectrodes present fast response times, good reversibility, and near-Nernst sensitivity.
